# Supplementary material for: Imaging With Nature: Compressive Imaging Using a Multiply Scattering Medium
Source: Sci Rep. 2014 Jul 9;4:5552. doi: 10.1038/srep05552 (PMC4087920; doi:10.1038/srep05552)
Supplement: Supplementary Information [file srep05552-s1.doc]

Imaging With Nature: Compressive Imaging Using a Multiply
Scattering Medium

**Antoine Liutkus^1^, David Martina^1^, Sébastien Popoff^1^, Gilles Chardon^1,2^, Ori Katz^1^, Geoffroy Lerosey^1^, Sylvain Gigan^1^, Laurent Daudet^1^, Igor Carron^3^**

^1^Institut Langevin, ESPCI ParisTech, Paris Diderot Univ., UPMC Univ. Paris 6, CNRS UMR 7587, Paris, France

^2^Acoustics Research Institute, Austrian Academy of Sciences, Vienna.

^3^TEES SERC, Texas A&M University.

Supplementary material

**Experimental setup**

**
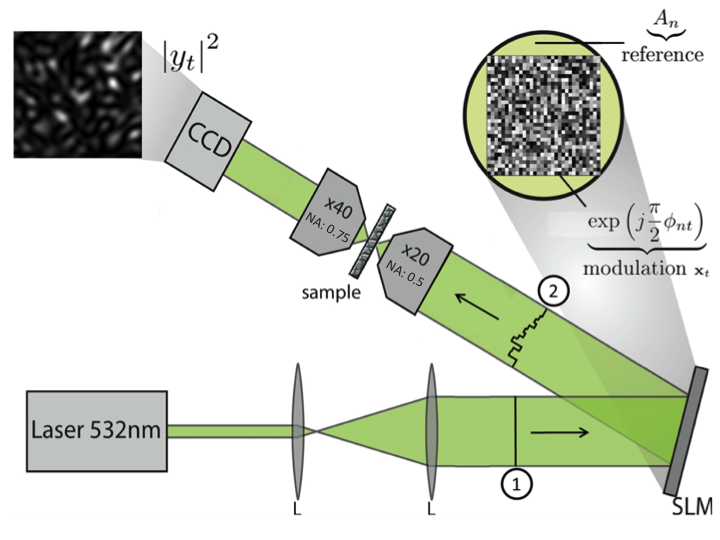
**

**Fig. S1**  Experimental setup for light diffusion through a scattering medium (24). The coherent wavefront from a laser is modulated using a SLM and transmitted through the medium. A CCD camera measures the wavefront at the output of the medium

For one particular measurement $t$, the experiment is displayed on figure S1. A laser beam is enlarged by a couple of (L-L) lenses and the resulting wavefront (1 on figure S1) is partly reflected by a Spatial Light Modulator (SLM) and partly by its support, respectively leading to the modulated wavefront$x_{t}$ and the reference wavefront$A$, which is constant over $t$. The SLM is composed of a 32 x 32 matrix of $N=1024$ segmented micromirrors (Kilo-DM, Boston Micromachines Corporation). Each $n$ of those mirrors is a 300μm square and locally controls the phase of . Both $x_{t}$ and $A$ combine to form the reflected wave (2 on figure S1), whose value at position $n$ is thus $A_{n}x_{nt}$. This wave is then propagated through an opaque 300μm-thin layer of compacted zinc oxide (ZnO) powder. In this medium, light is reflected many times and finally collected and imaged by a CCD camera. The interested reader is referred to (19,24) for more details concerning the experimental setup. In all the remaining of this study, when we mention a specific number $M$ of measurements, we experimentally refer to a subset of the CCD pixels. Since those pixels are always randomly chosen, the corresponding measurements are equivalent to those performed by $M$ arbitrarily located sensors.

The complex wave $y_{mt}$ at each of the $m=1\ldots M$ output positions is estimated using 4 intensity measurements done by the CCD camera thanks to the phase-stepping technique, which is detailed in (41,46). Note that in all the following, each complex measurement is hence understood as a combination of 4 intensity measurements. In further studies, *compressive phase retrieval* techniques (30) may be used to directly process intensity measurements $|y|$ instead of $y$ and hence further reduce the acquisition time. For now, we will simply consider the complex output$y$, which are related to $Ax_{t}$ by:

$$y_{mt}=\sum_{n=1}^{N} \bar{H}_{mn}A_{n}x_{nt},$$

where$\bar{H}$ is the intrinsic TM of the scattering media. Since the reference wave $A$ is kept fixed during the whole process thanks to the stability of the laser beam, it can safely be merged with $\bar{H}$ so as to yield

$$H_{mn}=\bar{H}_{mn}A_{n},$$

which will subsequently be called the TM, although it depends both on the scattering medium and the laser input. The slight abuse of notation is largely justified by the very high stability of lasers available on the market. Its estimation rather than $\bar{H}$ is sufficient to proceed to compressed sensing using scattering media. Note however that since $A_{n}$ stays constant over different measures $t$, it is possible through calibration to identify $\bar{H}$ from $H$ (24,25).In any case, the complex output $y$ is given by:

$$y_{mt}=\sum_{n=1}^{N} H_{mn}x_{nt},$$

which can be written in a more compact matrix-form as $Y=HX$, where $Y=[y_{mt}]_{m,t}$ and

$X=[x_{nt}]_{n,t}$are$M\times T$ and $N\times T$ matrices, respectively, whereas $H$ is the (complex) $M\times N$TM.

**Estimation of the Transmission Matrix**

In (24,25), Popoff et al. propose to estimate the TM $H$ using an orthonormal basis as input and hence having $X$ as a $N\times N$ matrix. The choice of the Hadamard basis to this purpose is judicious since all its entries are $\pm1$, which leads to $x_{nt}=\pm j$. Therefore, if $B_{N}$ denotes the $N\times N$Hadamard basis, the measured matrix $Y$ is $Y=HB_{N}$. If $I_{N}$ is defined as the $N\times N$ identity matrix, one of the properties of $B_{N}$ is to be its own inverse, leading to $B_{N}B_{N}=I_{N}$ and hence $H=YB_{N}$. This very simple procedure leads to a straightforward estimation of the TM $H$.

However, a better estimation of the TM is possible, provided more calibration measures are done, i.e. by choosing $T>N$. In that case, $Y=HX$still holds but $X$ is not an orthonormal basis nor its own inverse. However, $H$ can still be estimated straightforwardly through Least-Squares as:

$$\hat{H}=YX^{H}\left(XX^{H}\right)^{-1}$$

where denotes Hermitian (conjugate) transpose. This formula is actually a special case of a much more general setting, where noisy observations are accounted for and where estimation of the TM is performed through Least-Squares estimation.

In any case, in our experimental setup, instead of using a single $N\times N$Hadamard matrix as$X$, the input matrix $X$ for calibration was built as $X=\exp\left(j\Phi\right)$, with $\Phi$ being the horizontal concatenation of $B_{N}$ and a large random $N\times5N$ matrix with independent entries, uniformly distributed on the interval $\left[0 ; 2\pi\right]$. Then, after measurements have been performed, $H$ is estimated through the formula above.

A clear limitation of the approach is that estimation of the TM requires the linear outputs $Y=HX$. Even if these linear outputs may be obtained using phase-stepping techniques in several wavelengths, there are scenarios where only their intensity may be available. More sophisticated techniques (21) may be used in that case to estimate the TM using such measurements, with good performance in practice. Such approaches are the topic of current work.

**Virtually sparse intensity inputs**

In this section, we describe how the input data to the proposed imaging system was generated. S*parse* signals are zero most of the time and only scarcely nonzero. However, due to the particular experimental setup, where light is modulated using a phase-only SLM, we cannot consider signals, which are sparse in the Dirac (canonical) domain. Indeed, this would amount to having $x_{nt}=0$ most of the time, which is impossible because all $x_{nt}$ have the same amplitude: our SLM performs *phase* and not *amplitude* modulation.

However, we can use a simple trick that was already considered by Popoff *et al.* in (41) to generate an arbitrary (virtual) phase and amplitude object from a phase modulator. We use the same technique to build *virtual sparse objects* that are constructed as follows. First, build a $N\times1$ random phase vector , called *reference*, and measure the corresponding $M\times1$ complex output $y_{r}$. Second, randomly choose $k$ entries in , called the *support* and set their values as new random phases to build the vector , which is identical to except for only $k$ entries. The corresponding complex output $y_{r}$ is measured, and thanks to the linearity of the optical propagation, the difference $y=y_{r}-y_{s}$ corresponds to the complex output of the system for the *sparse virtual input object*$x=x_{r}-x_{s}$. Using this procedure, we were able to measure the output of the system for sparse input vectors of arbitrary sparsity$k$.

We highlight the fact that this way to build sparse inputs is required only because we used a SLM to control the input wavefront and not because of intrinsic limitations of the imaging method we propose. On the contrary, we emphasize that such virtual measurements actually lead to additional (doubled) noise, making the imaging process only more difficult.

Additionally and as done in (41), we were able to measure $P=3$ several outputs corresponding to different *illuminations* of the same virtual sparse object. This was achieved by using the same support for $P$ different reference phases. In essence, we thus settle in the Multiple Measurement Vector paradigm, abbreviated as MMV (20) and depicted in figure S2. The total number of complex measurements for each trial is hence $3M$, used to estimate $3N$ values of the input field.

In our experiments, we repeated this procedure so as to build a very large number of virtual objects of varying sparsity, from $k=1$ to $k=N$, along with their corresponding outputs.

**Algorithm for reconstruction using compressed sensing**

Suppose for now that the considered input wave fronts$x$ are sparse in the canonical domain, thus being virtual objects in our experimental setup as described above. We suppose that their sparsity$k$ is known and that the complex outputs $Y$ of the system for $P$ different illuminations of the same object are available, as depicted on figure S2.

**Fig. S2** The$P$ measurements vectors of $Y$ correspond to the output of the system to $P$ different sparse inputs having the same support. For inputs that are sparse in a base $B$ different from the canonical one, $H$ is simply replaced by $HB$.

We measure the $M\times P$ matrix $Y$, corresponding to the concatenation of $P$ outputs and given by $Y=HX$, where $X$ is the unknown input matrix to estimate and $H$ is the TM. Provided $H$ has been estimated through calibration, any algorithm capable of estimating a sparse vector given $M$ random projections of it can be used for the purpose of estimating $X$. In our experiments, we made use of basic multichannel Orthogonal Matching Pursuit, OMP (42), which is straightforward to implement. Even if more sophisticated methods may be used, we purposefully focused on the most simple and widely accepted approach to CS, since the purpose of this study was not to concentrate on alternative estimation methods, which may rather be the topic of future work. Note that when $P=1$, the whole procedure simply becomes equivalent to classical OMP.

Once the input $X$ has been estimated, the correlation of its support with ground truth is computed and estimation is said to be successful if this correlation lies above 0.9, meaning that at least 90% of the original support has been identified. When we are considering vectors that are not sparse in the Dirac (canonical) basis but in an alternative basis $B$, notably the Fourier basis as explained above, the same procedure can be applied using $HB$ instead of $H$ as a measurement matrix.

We applied this procedure for approximately 25000 different inputs, corresponding to a large range of sparsity$k$ from 1 to $N=1024$, and for many different values for the number $M$ of measurements, so as to yield a complete phase transition as found by Donoho and Tanner (31,43), displayed on Fig. 5. Each cell of this figure gives the average observed performance for the corresponding set of $\left(k,M\right)$ parameters over approximately 50 independent trials.

An identical experiment was then performed with measurements that are obtained by simply multiplying the sparse inputs by a synthesized i.i.d. Gaussian matrix and further adding a noise whose average amplitude is set to 17% of the observed average amplitude of the synthesized clean output. This matrix is estimated and used for CS in exactly the same manner as for the experimental data, in effect comparing performance of the presented imager with that of an idealized random sensor whose matrix would be unknown but estimated using noisy data. The transition curve for this idealized case is displayed in figure 4 as a dashed line.

**Fourier-sparse inputs**

Even if virtual objects are a good way to simulate objects, which are sparse in the canonical domain with arbitrary sparsity$k$, it is desirable to test the proposed imaging system using direct measurements of sparse objects. To this purpose, we measured the output of the system when the input $x$, of constant modulus, is sparse in the 2D-Fourier domain. In other words, it is easy to build $x$ as a 2D plane wave so that its modulus is constant while only one element of its Fourier transform is non-zero and corresponds to its wave number.

Although this procedure is simple, it is difficult to generalize it for arbitrary sparsity$k$, since it is not straightforward to build 2D wavefronts of constant modulus and arbitrary sparsity in the Fourier domain. Given one sparsity level (either $k=1$ or $k=20$), performance of the imaging method is evaluated as a function of the number $M$ of measurements and the results are displayed on Fig. S3.

**Fig. S3** Compressed sensing performance for the recovery of signals which are sparse in the Fourier domain. The two curves correspond to the probability of success as a function of the number of measurements$M$, when the unknown signal is either a plane wave ($k=1$) or the superposition of many planewaves ($k=20$). Each point is the average of 128 independent trials.

As can be seen on this figure, 15 measurements are sufficient to properly recover the input wavefront of the system, provided it is a planewave. This result demonstrates that the proposed imaging system is indeed universal and that its performance well matches results predicted by CS theory.

Supplementary reference

1. S. Tripathi, et al. "Vector transmission matrix for the polarization behavior of light propagation in highly scattering media." *Opt. Express* ***20.14*** (2012): 16067-16076.
